# Circulating sex hormones and risk of atrial fibrillation: A systematic review and meta-analysis

**DOI:** 10.3389/fcvm.2022.952430

**Published:** 2022-08-22

**Authors:** Peng Hu, Jun Huang, Yi Lu, Murui Zheng, Haiyi Li, Xueru Duan, Hai Deng, Wenjing Zhao, Xudong Liu

**Affiliations:** ^1^Department of Epidemiology, School of Public Health, Sun Yat-sen University, Guangzhou, China; ^2^School of Public Health, Guangdong Pharmaceutical University, Guangzhou, China; ^3^Department of Geriatrics, Institute of Geriatrics, Guangdong Provincial People’s Hospital, Guangdong Academy of Medical Science, Guangzhou, China; ^4^Health Effects Institute, Boston, MA, United States; ^5^Guangzhou Center for Disease Control and Prevention, Guangzhou, China; ^6^Shantou University Medical College, Shantou, China; ^7^Department of Cardiology, Guangdong Cardiovascular Institute, Guangdong Provincial People’s Hospital, Guangdong Academy of Medical Science, Guangzhou, China; ^8^School of Public Health and Emergency Management, Southern University of Science and Technology, Shenzhen, China

**Keywords:** sex hormones, atrial fibrillation, total testosterone, estradiol, dehydroepiandrosterone sulfate

## Abstract

**Background:**

Sex hormones are associated with many cardiovascular risk factors, but their effects on atrial fibrillation (AF) incidence remain unclear. This systematic review and meta-analysis aimed to evaluate the association of circulating sex hormones with AF risk by pooling available data from observational studies.

**Methods:**

A systematic literature search for pertinent articles with case-control and cohort designs was conducted *via* five databases up to 7 July 2021. A meta-analysis with six cohort studies was conducted separately on men and women. Adjusted relative risk (RR) with a 95% confidence interval (CI) was derived by comparing the highest with the lowest levels of a specific sex hormone and by using a random-effect or fixed-effect model. Heterogeneity was tested using the *I*^2^ statistic and the Q-test.

**Results:**

A total of six cohort studies and four case-control studies were included. In a meta-analysis of cohort studies, dehydroepiandrosterone sulfate (DHEAS) was associated with a decreased risk of AF in men (RR: 0.729, 95% CI: 0.559–0.952, *I*^2^ = 50.0%, *P*_–heterogeneity_ = 0.157) after combining results from two cohort studies; total testosterone was not associated with any risk of AF in men and postmenopausal women, and AF risk was not associated with estradiol in men after synthesizing available studies.

**Conclusion:**

This study indicates that a higher endogenous DHEAS level was associated with a lower AF risk in men, whereas total testosterone and estradiol were not associated with AF risk. Longitudinal studies with multiple monitoring are needed to further promulgate the relationship between various circulating sex hormones and AF risk.

## Introduction

Atrial fibrillation (AF) is the most common cardiac arrhythmia. Its incidence and prevalence increased during the past two decades and will continue to increase in the next 10 years (1, 2). AF is the common cause of cardioembolic stroke and heart-related hospitalizations, resulting in severe mortality and disability (3, 4). Studies from different regions suggested that the incidence of AF increased with advancing age and tended to be higher in men than in women (5–7); however, compared with men, women showed more severe atypical symptoms and poorer quality of life, as well as a higher risk of adverse events, such as stroke and death associated with AF (8, 9).

Gender-related endogenous sex hormones have gotten much more attention recently and were reported to be associated with cardiovascular risk factors, such as hypertension (10), obesity (11, 12), and type 2 diabetes mellitus (13, 14). Cardiac myocytes could express estrogen and androgen receptors, indicating that sex hormones might influence ion channels and their expression and finally affect the heart rhythm (15, 16). However, the current epidemiological evidence about the associations of sex hormones with the incidence of AF is inconsistent. For instance, Zeller et al. found that low serum total testosterone (TT) concentration was associated with an increased risk of AF in men (17), while Berger et al. reported a positive association of plasma testosterone level with the incidence of AF in men (18); estradiol produced from testosterone aromatization is linked to AF risk (19), but this was inconsistent with other studies (20–22).

Until now, the association of circulating sex hormones with AF risk remains unclear. Thereafter, we conducted this systematic review with meta-analysis in light of MOOSE guidelines (23) to examine the association between circulating sex hormones and AF risk.

## Materials and methods

### Search strategy and selection criteria

Up to 7 July 2021, five electronic databases, including PubMed, Embase, Web of Science, Chinese National Knowledge Infrastructure (CNKI), and WANFANG DATA, were searched. The following key words were used: “sex hormone,” “gonadal steroid hormones,” “estrogen,” “oestrogen,” “estradiol,” “oestradiol,” “progestins,” “progestagens,” “progestational hormones,” “progestogens,” “progesterone,” “androgen,” “testosterone,” “dehydroepiandrosterone sulfate,” “follicle stimulating hormone,” “luteinizing hormone,” “prolactin,” “androstenedione,” “sex hormone binding globulin,” “atrial fibrillation,” and “auricular fibrillation.” Details of the search strategy are shown in Supplementary Table 1. Citation and reference tracking through both Scopus and Web of Science were also used to identify studies.

Two researchers (PH and WZ) conducted the literature search independently, reviewed, and identified relevant literature. Disagreements were resolved by group discussions with the third researcher (XL). Studies that met the following criteria were included: (1) case-control studies or cohort studies; (2) the serum or plasm levels of TT, bioavailable testosterone (BT), estradiol, dehydroepiandrosterone sulfate (DHEAS), free dihydrotestosterone (DHT), and sex hormone binding globulin (SHBG) were detected; (3) published in English or Chinese; (4) effect estimates (including odds ratio, relative risk (RR), and hazard ratio) and 95% confidence intervals (95% CI) were reported or they can be calculated according to the reported data. Studies were excluded if they were duplicated studies, cross-sectional studies, case reports, animal studies, *in vitro* and *in vivo* experiments, reviews, editorials, comments, abstracts, effect estimates were not reported or could not be calculated, and studies in neither English nor Chinese.

### Data extraction

Detailed information from each study was extracted, and the information included the first author, publication year, study design, sample size, country in which the study was conducted, age, gender, sex hormone detection method, whether to take samples after fasting, whether to exclude subjects with hormone therapy, confounding factor for multivariable analysis, body mass index, median or mean of follow-up years for the cohort study, effect calculating approach, and specific sex hormone. The effect estimates and 95% CIs for each specific sex hormone obtained from the multivariable model were also extracted.

The quality of each study was assessed using the Newcastle-Ottawa Scale (NOS) criteria for the included cohort studies or case-control studies (24). The maximum total score is nine stars: four stars for selection, two stars for comparability, and three stars for assessment of outcomes or exposures. The studies were classified as high quality (7–9 stars), moderate quality (4–6 stars), and low quality (0–3 stars) in terms of the total scores.

### Statistical analysis

A systematic review was conducted among cohort and case-control studies, and the meta-analysis was only conducted among cohort studies as the cause-and-effect timing sequence cannot be determined among case-control studies. The RR was used to uniformly display the pooled effect of cohort studies in this study. A meta-analysis was conducted separately in men and in women to generate the pooled RR for each specific sex hormone. The pooled RR with 95% CI was derived by comparing the highest sex hormone levels with the lowest and by using a random-effect or fixed-effect model. The effect estimates in Magnani’s report (20) were calculated by comparing the lowest and highest categories; hence, the reciprocal of the original effect value was used when the pooled analysis was performed (25). Two articles (17, 20) reported the effect estimates of every one standard deviation change for each specific sex hormone; therefore, this kind of effect estimate was transformed to the effect obtained by comparing the highest with the lowest tertiles, through the approach suggested by Danesh et al. (26). These effect estimates used for pooled analysis were reckoned by multiplying 2.18 with the log-transformed RR (26). Magnani et al. (20) reported the RR stratified by age; thus, we first calculated the overall effect estimates by using the inverse variance method, and thereafter used these overall effect estimates when performing the meta-analysis.

The *I*^2^ statistic and the Q-test were used to examine the heterogeneity. The *I*^2^ value of more than 50% or *P*-value from the Q-test of less than 0.1 suggested significant heterogeneity (27). A random-effect model was adopted if there was significant heterogeneity; otherwise, a fixed-effect model was selected. To seek the sources of heterogeneity and to examine the robustness of the main results, subgroup analysis was conducted based on several study characteristics: region (United States, non-United States), age (<63 years, ≥63 years), body mass index (<27.5 kg/m^2^, ≥27.5 kg/m^2^), and TT assessment method (radioimmunoassay, others: liquid chromatography-tandem mass spectrometry, chemiluminescent microparticle immunoassay). A random-effects meta-regression analysis was also conducted to estimate these characteristics for the potential sources of heterogeneity in this study. Repeated analysis was conducted to examine the contribution of each study to the pooled estimates by omitting one study at a time. The publication bias was illustrated by using the funnel plot and Begg’s test. An asymmetric funnel plot or a *P*-value of Begg’s test of lower than 0.05 was considered to have significant publication bias. All statistical analyses were conducted using the Stata 12.0 software (Stata Corporation, College Station, TX, United States).

## Results

### Systematic review

**FIGURE 1:**
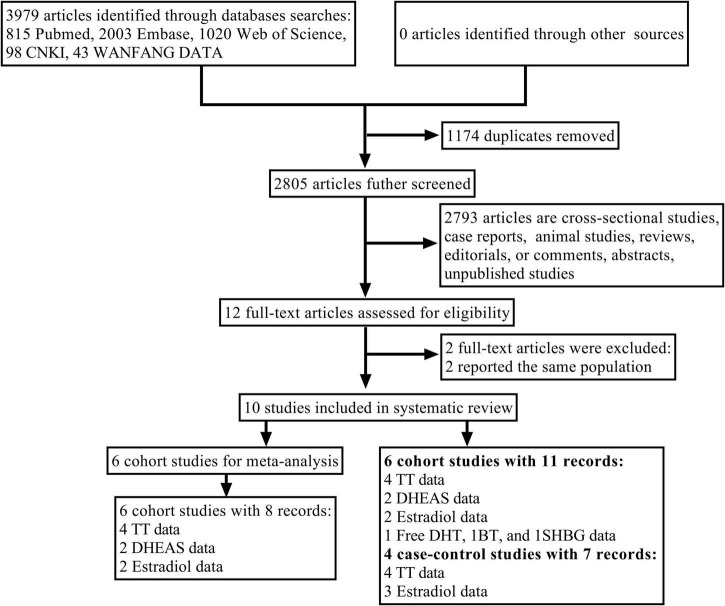
Flowchart of the study selection process. AF, atrial fibrillation; BT, bioavailable testosterone; DHEAS, dehydroepiandrosterone sulfate; free DHT, free dihydrotestosterone; SHBG, sex hormone binding globulin; TT, total testosterone.

The flowchart of study selection is shown in Figure 1. A total of 3,979 articles were searched from five databases. Approximately 1,174 articles were excluded for duplication, and 2,780 were excluded after the screening of titles and abstracts. Among the twelve articles retained for further full-text review, two articles focused on the same population and reported similar results to another two articles, respectively; hence, only two recently published articles were selected. Finally, six cohort studies (17, 18, 20, 21, 28, 29) and four case-control studies (22, 30–32) were included in this systematic review, and six cohort studies (17, 18, 20, 21, 28, 29) were selected for meta-analysis. The NOS was used to evaluate the quality of studies included (Supplementary Table 2). All selected studies had moderate to high quality, and the quality score for cohort studies ranged from 7 to 9, and for case-control studies, it ranged from 5 to 7.

The general characteristics of the 10 selected studies are shown in Tables 1, 2, and the main sex hormones measured in the included studies are shown in Tables 3, 4. All four case-control studies were conducted in China (22, 30–32); three studies focused on both estradiol and TT, and one study only focused on TT. For included cohort studies, four came from the United States (18, 20, 21, 28), one from the Netherlands (29), and one from Finland (17); four studies in men (17, 18, 20, 21) and three studies in menopausal women (17, 18, 21) focused on TT; two studies in men (20, 21) and one study in post-menopausal women (21) on estradiol; two studies in men on DHEAS (20, 29); one study in men on free DHT (28), one study on BT (21), and one study on SHBG (21); the median or mean of follow-up years of six cohort studies ranged from 9.5 to 13.8 years.

**TABLE 1:** General characteristics of the included cohort studies.

Reference, country	Study design	Total samples (men/ women), *n*	Outcome (men/ women), *n*	Age, years	Follow-up years	BMI	Morning blood samples	Whether to take samples after fasting	Whether to exclude subjects with hormones therapy	Circulating sex hormones detection method^a^	Effect estimator calculation method
Rosenberg et al. (28), United States	Longitudinal cohort	1,019 (1,019/–)	AF 304 (304/–)	76.3 ± 4.9	9.5 years	26.7 ± 3.7	Not mention	Yes	No	TT and DHT: liquid chromatography–tandem mass spectrometry assay; SHBG: time-resolved fluoro-immunoassay; free testosterone and free DHT: Mazer method	Cox proportional hazards regression model
Berger et al. (18), United States	Prospective cohort study: Atherosclerosis Risk in Communities (ARIC) study	9,282 (4,224/5,058)	AF 1490 (772/718)	All 63 (range 52–75)**** Male 63 ± 5.7**** Female 63 ± 5.4	13.7 years	Male 28 ± 4.5; female 29 ± 6.2	Yes	Not mention	No	TT: liquid chromatography tandem mass spectrometry	Cox regression models
Krijthe et al. (29), Netherlands	Population-based prospective cohort study (The Rotterdam Study)	1,180 (547/633)	AF 129 (67/62)	69 ± 8.4	12.3 years	26.1 ± 3.4	Not mention	No	No	DHEAS: coated tube radioimmunoassays	Cox proportional hazards analyses
Magnani et al. (20), United States	Prospective, community-based cohort study (The Framingham Heart Study)	1,251 (1,251/–)	AF 275 (275/–)	68.0 ± 8.2	10 years	26.7 ± 3.8	Not mention	Not mention	No	TT, estradiol, DHEAS: radioimmunoassays	Cox proportional hazards regression analyses.
O’Neal et al. (21), United States	Population-based prospective cohort study (Multi-Ethnic Study)	4,883 (3,003/1,880)	AF 613 (613/–)	All 63 ± 10**** Men 62 ± 10**** Women 65 ± 9.2	10.9 years	Male 28 ± 4.4; female 29 ± 6.1	Not mention	Yes	Yes	TT: RIA kits**** SHBG: chemiluminescent enzyme immunometric assay using Immulite kits**** E2: ultrasensitive RIA kit**** BT: [total T × (percent-free T × 0.01)]	Cox regression
Zeller et al. (17), Finland	Population-based prospective cohort study (the FINRISK study)	7,892 (3,876/4,016)	AF 426 and/or IST 276	Men 49.2 (range 37.7–60.7)**** Women 47.2 (range 36.5–57.6)	13.8 years	Male 26.6 (range 24.3–29.1); female 25.5 (range 22.7–29.1)	Not mention	Yes	Yes	TT: a chemiluminescent microparticle immunoassay (CMIA)	Cox regression

^a^AF, atrial fibrillation; BMI, body mass index; BT, bioavailable testosterone; CI, confidence interval; DHEAS, dehydroepiandrosterone sulfate; DHT, dihydrotestosterone; HR, hazard ratio; IST, ischemic stroke; RIA: radioimmunoassay; SHBG, sex hormone binding globulin; TT, total testosterone.

**TABLE 2:** General characteristics of the included case-control studies.

Reference, region	Study design	Sample size of cases	Sample size of controls	Age, years (case/control)	BMI (case/control)	Morning blood samples	Whether to take samples after fasting	Whether to exclude subjects with hormones therapy	Circulating sex hormones detection method	Effect estimator calculation method
Lian (30), Zhejiang, China	Hospital based case-control	100 men (50 paroxysmal AF/50 persistence AF)	130 men	Case: paroxysmal AF 52.68 ± 7.16; persistence AF 55.40 ± 5.79**** Control: 49.54 ± 5.45	Case: paroxysmal AF 24.12 ± 1.88; persistence AF 24.16 ± 2.02**** Control: 23.98 ± 2.70	Not mention	Yes	No	Chemiluminescence method	Logistics regression analysis
Wei (32), Gansu, China	Hospital based case-control	63 men	76 men	Case:**** 66.90 ± 9.66**** Control: 65.86 ± 9.69	Case:**** 24.55 ± 2.74**** Control: 24.83 ± 2.66	Yes	Yes	Yes	Chemiluminescence method	Logistics regression analysis
Ma (31), Gansu, China	Hospital based case-control	107 women	72 women	Case: 70.50 ± 7.50**** Control: 66.07 ± 8.10	Case: 24.63 ± 3.79**** Control: 24.08 ± 3.12	Yes	Yes	Yes	Chemiluminescence method	Logistics regression analysis
Lai et al. (22), Zhejiang, China	Hospital based case-control	58 men	58 men	Case: 46.1 ± 9.7**** Control: 45.2 ± 8.6	Case: 25.1 ± 2.6**** Control: 24.8 ± 2.8	Not mention	Not mention	No	Radioimmunoassay	Logistics regression analysis

AF, atrial fibrillation; BMI, body mass index.

**TABLE 3:** Main sex hormones measured in the included cohort studies.

Reference, country	Exposure details^d^	Confounding factor for multivariable analyses	Specific sex hormone	Effect estimator					Transformation of effect estimator
					Men		Women		
					HR (95% CI)	*P*-value	HR (95% CI)	*P*-value	
Rosenberg et al. (28), United States	Low free DHT (<0.16 ng/dl); the lowest quintile of values	Demographics (race, education, income, clinic location, and smoking status), clinical risk factors (BMI, standing height, DM, use of antihypertensive medications, SBP (mm Hg), depressed LV function, serum cystatin C level, and use of loop diuretics.), center atrial diameter, and serum NT-proBNP levels	Free DHT	Q1 Q2–Q5	1.48 (1.01–2.17) 1	0.044 –	– –	– –	– –
Berger et al. (18), United States	Testosterone levels were split into sex-specific quartiles:**** Men:**** Q1: ≤388.62 ng/dl**** Q2: 388.63–511.23 ng/dl**** Q3: 511.24–659.25 ng/dl**** Q4: >659.25 ng/dl**** Women:**** Q1: ≤17.20 ng/dl**** Q2: 17.21–23.17 ng/dl**** Q3: 23.18–31.55 ng/dl**** Q4: >31.55 ng/dl	Race, age, BMI, smoking status, estimated glomerular filtration rate, SBP, antihypertensive medication use, diabetes, prevalent coronary heart disease, and prevalent heart failure	Total testosterone	Q1 Q2 Q3 Q4	1 1.21 (0.99–1.48) 1.17 (0.95–1.45) 1.33 (1.07–1.66)	– – – –	1 1.04 (0.84–1.29) 1.07 (0.87–1.33) 0.99 (0.80–1.22)	– – – –	– – – –
Krijthe et al. (29), Netherlands	DHEAS quartiles**** Q1: 0.01–1.73 mmol/L**** Q2: 1.74–2.95 mmol/L**** Q3: 2.96–4.74 mmol/L**** Q4: 4.75–23.08 mmol/L	Age, sex, SBP, diastolic blood pressure, blood pressure lowering therapy, BMI, total and high density lipoprotein (HDL) cholesterol, smoking status, alcohol use, sex hormone therapy, prevalent myocardial infarction, heart failure and DM at baseline, and carotid plaque score	DHEAS	Q1 Q2 Q3 Q4	1 0.88 (0.46–1.70) 0.68 (0.34–1.39) 0.42 (0.19–0.96)	– – – –	1 0.74 (0.39–1.32) 0.67 (0.34–1.34) 0.33 (0.14–0.80)	– – – –	– – – –
Magnani et al. (20), United States	Testosterone: a standard clinical threshold, 300 ng/dl per SD decrease	Smoking, BMI, SBP, hypertension treatment, diabetes mellitus, PR interval, significant murmur, and prevalent heart failure	Total testosterone Estradiol DHEAS	Age 55–69 years Age 70–79 years Age≥80 years Per SD decrease Per SD decrease	1.30 (1.07–1.59) 1.14 (0.91–1.44) 3.53 (1.96–6.37) 1.12 (1.01–1.26) 1.12 (0.99–1.28)	0.008 0.26 <0.0001 0.04 0.08	– – – – –	– – – – –	Combined 0.628 (0.463–0.894)^abc^ 0.78 (0.60–0.98)^bc^ 0.78 (0.58–1.02)^bc^
O’Neal et al. (21), United States	TT: T3 vs. T1**** BT: T3 vs. T1**** Estradiol: T3 vs. T1**** SHBG: T3 vs. T1	Age, race, education, income, current smoking, study site, diabetes, systolic blood pressure, height, BMI, aspirin, antihypertensive medications, lipid-lowering therapies, and center ventricular hypertrophy	Total testosterone BT Estradiol SHBG	T1 T2 T3 T1 T2 T3 T1 T2 T3 T1 T2 T3	1 1.12 (0.87–1.45) 1.20 (0.93–1.55) 1 0.96 (0.75–1.74) 1.32 (1.01–1.74) 1 0.85 (0.66–1.09) 1.09 (0.84–1.40) 1 1.05 (0.79–1.39) 1.04 (0.78–1.39)	– 0.36 0.16 – 0.71 0.044 – 0.19 – 0.73 0.77	1 0.99 (0.72–1.38) 0.96 (0.70–1.32) 1 0.96 (0.69–1.32) 0.81 (0.58–1.13) 1 0.89 (0.64–1.23) 0.84 (0.60–1.16) 1 1.34 (0.94–1.91) 1.39 (0.96–1.99)	– 0.98 0.8 – 0.79 0.22 – 0.47 0.28 – 0.10 0.079	– – – – – – – – – – – –
Zeller et al. (17), Finland	TT per one nmol/L increase	Age was used as time-scale, adjusted for geographical region, total cholesterol (log), HDL-C (log), systolic blood pressure (log), hypertension medication, known diabetes, smoking status, waist-hip-ratio, and time of day of the blood draw	Total testosterone	TT per one nmol/l increase	0.98 (0.97–1.00)	0.049	1.17(1.02–1.36)	0.031	Men: 0.957 (0.936–1.000)^b^**** Women: 1.41 (1.04–1.95)^b^

^a^The overall effect estimates were calculated using the inverse variance method.

^b^This effect estimates used for pooled analysis were reckoned by multiplying 2.18 with the log-transformed relative risk.

^c^The reciprocal of the original effect value was used to calculate the effect estimate by comparing the highest and lowest categories.

BMI, body mass index; BT, bioavailable testosterone; CI, confidence interval; DHEAS, dehydroepiandrosterone sulfate; DHT, dihydrotestosterone; DM, diabetes mellitus; HDL-C, high-density lipoprotein cholesterol; HR, hazard ratio; IST, ischemic stroke; LV, left ventricular; NT-proBNP, N-terminal pro-B-type natriuretic peptide; RIA, radioimmunoassay; SBP, systolic blood pressure; SHBG, sex hormone binding globulin; TT, total testosterone.

**TABLE 4:** Main sex hormones measured in the included case-control studies.

Reference, region	Confounding factor for multivariable analyses	Specific sex hormone	Result			
			Men		Women	
			OR (95% CI)	*P*-value	OR (95% CI)	*P*-value
Lian (30), Zhejiang, China	Age, LAD, Hs-CRP	Total testosterone	0.992 (0.989–0.996)	<0.001	–	–
Wei (32), Gansu, China	Age, LAD, UA, Hcy	Total Testosterone	0.995 (0.991–1.000)	0.040	–	–
		Estradiol	0.969 (0.924–1.016)	0.193	–	–
Ma (31), Gansu, China	Age, BMI, LAD, BUN, creatinine, UA, Hcy, Cys-C, TC, TG, LDL-C	Total Testosterone	–	–	1.028 (0.992–1.066)	0.130
		Estradiol	–	–	0.957 (0.878–1.044)	0.322
Lai et al. (22), Zhejiang, China	Age, BMI, Systolic blood pressure, Diastolic blood pressure	Total Testosterone	1.003 (0.999–1.007)	0.104	–	–
		Estradiol	1.000 (0.943–1.061)	0.996	–	–

AF, atrial fibrillation; BMI, body mass index; BUN, blood urea nitrogen; CI, confidence interval; Cys-c, cystatin C; Hcy, homocysteine; Hs-CRP, high-sensitive C-reactive protein; LAD, left atrial diameter; LDL-C, low-density lipoprotein cholesterol; OR, odds ratio; TC, total cholesterol; TG, triglyceride; UA, serum uric acid.

For all ten studies, the average BMI in men ranged from 24.12 to 28.00 kg/m^2^, and the average BMI in post-menopausal women varied from 24.08 to 29.00 kg/m^2^. Among them, four studies (17, 21, 31, 32) excluded the individuals receiving sex hormone therapy, whereas the other six (18, 20, 22, 28–30) never did that. For the detection of sex hormones, three cohort studies (17, 21, 28) and three case-control studies (22, 31, 32) collected fasting blood samples. Circulating TT concentration was detected by using radioimmunoassay in three studies (20–22), chemiluminescent microparticle immunoassay in four studies (17, 30–32), and liquid chromatography-tandem mass spectrometry assay in one study (28). Estradiol concentration was measured by radioimmunoassay in three studies (20–22) and the chemiluminescence method in two studies (31, 32). DHEAS concentration was measured by radioimmunoassay in both studies (20, 29).

Confounders adjusted in each study were different. Age was used as a confounding factor in eight studies (17, 18, 21, 22, 29–32), and stratified analysis was conducted by age in two studies (20, 28). Body mass index was used as a confounding factor in seven studies (18, 20–22, 28, 29, 31), while the other three (17, 30, 32) did not do this. All six cohort studies used the smoking status as a confounding factor, but four case-control studies did not use smoking status as a confounding factor.

Among the included cohort studies that revealed the associations between TT level and AF risk, in men, one study showed that TT level was associated with increased AF risk (18), two studies showed TT level was associated with decreased risk of AF (17, 20), and one study did not find a significant association between TT level and AF risk (21); in post-menopausal women, two studies found TT level was not significantly associated with AF risk (18, 21), and one study showed that TT level was positively associated with AF risk (17).

The association between estradiol and DHEAS level with AF risk was revealed in two cohort studies, respectively. In men and post-menopausal women, one study showed that estradiol level was not significantly associated with AF risk (21), and in men, one study showed that estradiol level was associated with a lower risk of AF (20). In men and post-menopausal women, one study showed that DHEAS was associated with a decreased risk of AF (29), while one study showed that DHEAS level was not significantly associated with AF risk in men (20). In addition, one study showed that a low level of DHT was associated with an increased risk of AF risk in men (28); one study found that BT level was associated with increased AF risk in men, whereas no association was observed in post-menopausal women (21); one study showed that SHBG was not associated with any AF risk in men and women (21).

Among the included case-control studies, two studies showed that testosterone level was associated with a decreased AF risk in men (30, 32), and one study did not show an association between testosterone level and AF risk in men (22); two studies showed estradiol level was not significantly associated with AF risk in men (22, 32). Only one case-control study was conducted in post-menopausal women and did not find a significant association between testosterone and estradiol levels with AF risk (31).

### Meta-analysis of cohort studies

#### Total testosterone level and atrial fibrillation risk in men

**FIGURE 2:**
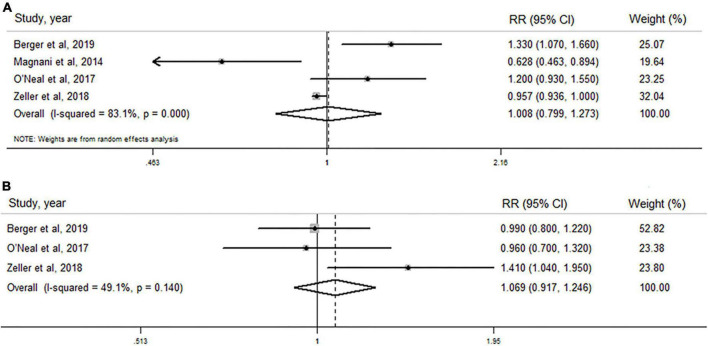
Forest plots of the association between total testosterone level and risk of atrial fibrillation in men and post-menopausal women by pooling cohort studies. **(A)** The random-effect model was used to assess the association between total testosterone level and risk of atrial fibrillation in men; **(B)** fixed-effect model was used to assess the association between total testosterone level and risk of atrial fibrillation in post-menopausal women.

As shown in Figure 2A, TT concentration was not associated with the risk of AF in men when results from four original studies were synthesized (RR: 1.008, 95% CI: 0.799–1.273, *I*^2^ = 83.1%, *P*-_heterogeneity_ < 0.001). Similarly, no significant association was found in any other subgroups stratified by regions, age, body mass index, and TT detection method (Table 5). Sensitivity analysis was repeated four times by omitting one study each time, and no significant change in the pooled results was found (Supplementary Figure 1a and Supplementary Table 3).

**TABLE 5:** Subgroup analysis for the association of total testosterone and atrial fibrillation risk in men.

Subgroups^a^	No.	Pooled RR (95% CI)	*P*_*h*_ ^b^	*I*^2^ (%)
All	4	1.008 (0.799–1.273)	0.000	83.1
Region				
United States	3	1.017 (0.674–1.533)	0.001	86.2
Non-United States	1	0.957 (0.926–0.989)	–	–
Age				
<63 years	2	1.033 (0.838–1.274)	0.085	66.3
≥63 years	2	0.923 (0.443–1.926)	0.000	92.8
BMI				
<27.5 kg/m^2^	2	0.801 (0.533–1.205)	0.013	84.0
≥27.5 kg/m^2^	2	1.273 (1.078–1.504)	0.549	0.0
TT assessment				
RIA	2	0.876 (0.464–1.651)	0.002	89.2
Others	2	1.107 (0.804–1.526)	0.004	88.2

^a^BMI, body mass index; RIA, radioimmunoassay; others, liquid chromatography-tandem mass spectrometry, chemiluminescence method, chemiluminescent microparticle immunoassay.

^b^*P*_*h*_, *P*-value from the Q-test.

#### Total testosterone level and atrial fibrillation risk in post-menopausal women

As shown in Figure 2B, TT level was not associated with AF risk in post-menopausal women when all three studies were pooled (RR: 1.069, 95% CI: 0.917–1.246), with lower heterogeneity (*I*^2^ = 49.1%, *P*_–heterogeneity_ = 0.140). In sensitivity analysis, the pooled analysis was repeated three times by excluding one study each time, and no significant change was observed (Supplementary Figure 1b and Supplementary Table 3).

#### Estradiol level and atrial fibrillation risk in men

**FIGURE 3:**
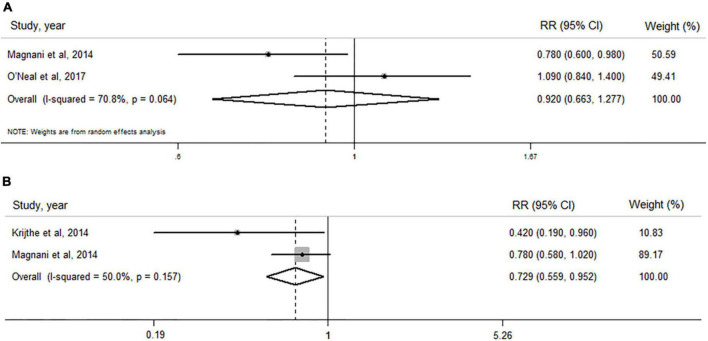
Forest plots of the association between levels of estradiol and dehydroepiandrosterone sulfate and risk of atrial fibrillation by pooling cohort studies. **(A)** The random-effect model was used to assess the association of estradiol level with atrial fibrillation risk in men; **(B)** the fixed-effect model was used to assess the association of dehydroepiandrosterone sulfate level with atrial fibrillation risk in men.

As shown in Figure 3A, no significant association of circulating estradiol levels with AF risk in men was observed when both studies were pooled (RR: 0.920, 95% CI: 0.663–1.277, *I*^2^ = 70.8%, *P*_–heterogeneity_ = 0.064).

#### Dehydroepiandrosterone sulfate level and atrial fibrillation risk in men

When two cohort (20, 29) studies including 2,431 subjects and 404 AF cases were pooled (Figure 3B), a high level of circulating DHEAS was significantly associated with a decreased AF risk (RR: 0.729, 95% CI: 0.559–0.952), with a lower heterogeneity (*I*^2^ = 50.0%, *P*_–heterogeneity_ = 0.157).

#### Publication bias

**FIGURE 4:**
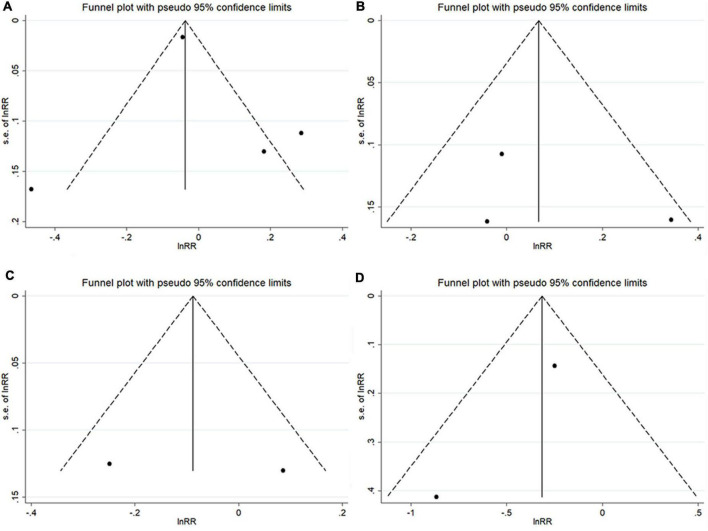
Funnel plots with pseudo 95% confidence limits among cohort studies. **(A)** The total testosterone level and atrial fibrillation risk in men; **(B)** total testosterone level and atrial fibrillation risk in post-menopausal women; **(C)** estradiol concentration and atrial fibrillation risk in men; and **(D)** dehydroepiandrosterone sulfate concentration and atrial fibrillation risk in men.

The funnel plots did not show any significant asymmetry (Figure 4). The Begg’s test did not reveal any significant publication bias when examining the associations between TT levels and AF risk in men (*P* = 0.734) and in post-menopausal women (*P* = 1.000), the associations between estradiol levels and AF risk in men (*P* = 1.000), and the association between DHEAS levels and AF risk in men (*P* = 1.000).

## Discussion

To the best of our knowledge, this systematic review and meta-analysis comprehensively examined the association of AF risk with circulating sex hormones. Based on the synthetization of limited evidence, the pooled results suggested that a higher level of DHEAS was associated with a lower risk of AF in men, whereas the association of AF risk with TT and estradiol was observed in neither men nor post-menopausal women. Similarly, the risk of AF was not associated with serum estradiol in men.

The pooled results in this study indicated that a high DHEAS level was associated with a 27.1% reduced AF risk in men. A negative association between DHEAS level and the risk of heart failure and mortality in both genders was found in a cohort study with 8,143 participants (33). Lower DHEAS concentration was also found to be associated with a higher risk of cardiovascular mortality (34, 35) and prognosis (36). Besides, DHEAS was inversely associated with sex-dependent diverse carotid atherosclerosis (37–39), and subjects with carotid atherosclerosis were at high risk of developing AF (40). The possible mechanisms may be due to the reason that DHEAS has the property of anti-inflammation (41, 42), which can influence the occurrence of AF (43). A pathological study has shown that the zona reticularis, responsible for most DHEAS production, seems to be more vulnerable to vascular damage (44). DHEAS can inhibit vascular remodeling by reducing neointima formation after vascular injury, thereafter reducing vascular inflammation (38).

The evidence investigating the association between TT level and the risk of AF was contradictory, no matter in the included cohort studies or case-control studies (17, 18, 20–22, 30, 32). The meta-analysis of cohort studies showed that AF risk was not associated with TT concentration in men and post-menopausal women, respectively, and AF risk was not associated with estradiol concentration in men in all two case-control studies and one cohort study (21, 22, 32). Similarly, other studies with meta-analysis did not demonstrate any significant association of TT and estradiol levels with cardiovascular disease risk in men and post-menopausal women (19, 45, 46). The Framingham Heart Study found that older men with decreased testosterone levels were at an increased AF risk, while Berger et al. found that a higher TT level was associated with an increased risk of AF in men, and the Multi-Ethnic Study of Atherosclerosis (MESA) found that TT level was not associated with AF risk in men. The different results of the studies may be related to the variation in the study populations examined among the studies; the men in the Framingham Heart Study were Caucasian and much older, while the Atherosclerosis Risk in Communities (ARIC) study and the MESA included populations of different races. Some important factors in the included studies may also cause the discrepancies, such as different numbers of included participants, the age difference of the participants in the studies, and various follow-up periods in the cohort studies. A recent study showed that after testosterone replacement therapy, the normalization of testosterone levels is associated with decreased AF incidence (47). However, the heterogeneity was relatively high among different studies, so more longitudinal studies with multiple monitoring or studies that include AF as an endpoint in clinical trials are needed to promulgate the association of AF incidence with TT and estradiol concentration.

There were insufficient studies to examine the association between the level of DHT, BT, and SHBG and AF risk. In the Cardiovascular Health Study in the United States with a median follow-up of 9.5 years, Rosenberg et al. found that men with serum-free DHT of less than 0.16 ng/dl were associated with a 1.48-fold risk of AF in men when compared with those with higher levels of DHT (28). By using data from the MESA in the United States and with a median follow-up of 10.9 years, O’Neal et al. examined the roles of serum levels of estradiol, BT, and SHBG in the development of AF (21); when comparing the highest with the lowest tertiles, serum BT was associated with a 32% increased risk of AF in men, whereas no association was observed in post-menopausal women (21); serum estradiol was not associated with AF risk in men and post-menopausal women (21); serum SHBG was not associated with any AF risk in men and women (21). Magnani et al. found that a 1-SD decrease in estradiol was associated with an increased risk of AF in men (20). Different sample sizes, age differences, and different confounding factors that the studies considered may lead to different results. However, these results should be verified in more studies with a delicate and rigorous longitudinal design.

The potential pathophysiology underlying the association between sex hormones and AF is complex. In rats, androgens depress spontaneous depolarization during atrial pacemaking, resulting in acute vasodilation, increased contractility, and an increased sino-atrial recovery time (48). Androgens can lead to a larger response to inotropes in male atria by a post-synaptic increase in intracellular cAMP and are independent of beta-1 adrenoceptors (49). Estrogens are those that drive the sensitivity to catecholamine by downregulating the beta-1 receptors depending on the estrous cycle, in female mice (50). Animal experiments showed that the increased release of atrial natriuretic peptide that resulted from cardiac overload was reduced by testosterone (51). Altered atrial electrophysiology may also provide a possible mechanism for the association between sex hormones and AF. Animal models have shown that atrial electrophysiology is changed *via* experimental modification of systemic hormones. Compared with controls, orchiectomized rats demonstrated greater repetitive atrial responses with left atrial pacing (52).

There were some limitations in the included studies. Almost all the included studies assessed the levels of circulating sex hormones at a single point in time; however, the longitudinal changes in the circulating level of sex hormones and whether these changes influenced the incidence of AF were unknown. The study by Berger et al. cannot avoid the effects of asymptomatic AF cases that may be missed in an outpatient setting, and false-negative misclassification may have occurred (18). Magnani et al.’s study (20) focused on middle-aged to older men in Europe, which cannot offer generalizability to younger people, women, and other races. The findings of O’Neal et al. (21) and Zeller et al. (17) did not include some important risk factors such as left atrial diameter and thyroid function, which may influence the results. The studies by Zeller et al. (17) and Berger et al. (18) only measured the levels of TT; however, TT includes SHBG and free testosterone, and the change in SHBG levels may cause the change in TT levels. The confounding factors considered in each included study were different, which may also cause the relatively high heterogeneity across the studies. For example, Krijthe et al. (29) and Zeller et al. (17) took total cholesterol and high-density lipoprotein cholesterol as two major confounding factors, while the other studies did not; Magnani et al. (20) considered PR interval and significant murmur as potential confounders, while the others did not; Berger et al. (18) included estimated glomerular filtration rate as a potential confounding factor, while the others did not.

The advantage of this study is that different sex hormones were fully considered, and for the first time, a qualitative systematic review with a quantitative meta-analysis was conducted to reveal the relationship between sex hormones and AF. The meta-analysis and sensitivity analysis were performed successively, and the results indicated the robustness and stability of the main results. Nevertheless, some limitations should be acknowledged in our study. First, specific sex hormones were measured by different methods, which may lead to information bias and thus may affect the consistency of the results; however, sensitivity analysis was repeated by omitting one study each time, and no significant change in pooled results was observed. Second, this study did not differentiate the AF types when doing the meta-analysis, so the effect of sex hormones on different types of AF needs further evaluation. Third, there were only six studies included; only two studies could be pooled to reveal the association of DHEAS and estradiol with AF risk in men, respectively; pooling results from limited evidence may influence the stability of our results to draw strong conclusions on the association between sex hormones and AF risk. Fourth, the potential heterogeneity among the included studies may exist in terms of population source, sample size, and subject characteristics; however, due to the limited data, the subgroup analysis and meta-regression based on these factors could not be performed for TT in women, estradiol in women, and DHEAS in men.

## Conclusion

The findings of this systematic review and meta-analysis indicate that a higher endogenous DHEAS level was associated with a lower AF risk in men, whereas no association was observed between TT and estradiol concentration and AF risk. Longitudinal studies with multiple monitoring are needed to further promulgate the relationship between various circulating sex hormones and AF risk.

## Data availability statement

Publicly available datasets were analyzed in this study. The data is available by contacting the corresponding authors or extracting from the original published research.

## Author contributions

XL and HD conceived, designed, and supervised the study. PH and WZ searched the data. PH analyzed the data. PH and JH drafted the manuscript. PH, MZ, HD, WZ, HL, YL, and XL reviewed and interpreted the findings. All co-authors provided comments and approved the final version.
