# Supplementary material for: Circulating sex hormones and risk of atrial fibrillation: A systematic review and meta-analysis
Source: Front Cardiovasc Med. 2022 Aug 22;9:952430. doi: 10.3389/fcvm.2022.952430 (PMC9441879; doi:10.3389/fcvm.2022.952430)
Supplement: Supplementary file 1 [file Data_Sheet_1.pdf]

Supplementary Material

- Table S1. Search strategy of the study.
- Table S2. Quality assessment of the included cohort.
- Table S3. Sensitivity analysis on the association of atrial fibrillation risk with total testosterone levels in men and women by omitting one study in a time.
- Figure S1. Sensitivity analyses on the association between levels of total testosterone and risk of atrial fibrillation.

Table S1. Search strategy of the study

	Pubmed/ Embase/ Web of Science search	CNKI/ WANFANG DATA search	Search
	(English database)	(Chinese database)	
1	Gonadal Steroid Hormones	性腺类固醇激素	Title/Abstract
2	Sex hormone	性激素	Title/Abstract
3	Estrogen	雌激素	Title/Abstract
4	Oestrogen	雌激素	Title/Abstract
5	Estradiol	雌二醇	Title/Abstract
6	Oestradiol	雌二醇	Title/Abstract
7	Progestins	孕激素	Title/Abstract
8	Progestagens	孕激素	Title/Abstract
9	Progestational Hormones	孕激素	Title/Abstract
10	Progestogens	孕酮	Title/Abstract
11	Progesterone	黄体酮	Title/Abstract
12	Androgen	雄激素	Title/Abstract
13	Testosterone	睾酮	Title/Abstract
14	Dehydroepiandrosterone sulfate	硫酸脱氢表雄酮	Title/Abstract
15	Follicle stimulating hormone	促卵泡刺激素	Title/Abstract
16	FSH	促卵泡刺激素	Title/Abstract
17	Luteinizing hormone	黄体生成素	Title/Abstract
18	LH	黄体生成素	Title/Abstract
19	Prolactin	催乳素	Title/Abstract
20	Androstenedione	雄（固醇）烯二酮	Title/Abstract
21	Sex hormone binding globulin	性激素结合蛋白	Title/Abstract
22	SHBG	性激素结合蛋白	Title/Abstract
23	Atrial Fibrillation	心房颤动	Title/Abstract
24	Auricular Fibrillation	心房纤维颤动	Title/Abstract
25	AF	房颤	Title/Abstract
26	1 OR 2 OR 3 OR 4 OR 5 OR 6 OR 7 OR 8 OR 9 OR10 OR 11 OR 12 OR 13 OR 14 OR 15 OR 16 OR 17 OR 18 OR 19 OR 20 OR 21 OR 22		
27	23 OR 24 OR 25		
28	26 AND 27		
	Database Searched:<br>Pubmed (https://pubmed.ncbi.nlm.nih.gov/)<br>Embase (https://www.embase.com/)<br>Web of Science (http://apps.webofknowledge.com/)<br>CNKI (https://cnki.net/)<br>WANFANG DATA (http://www.wanfangdata.com.cn/index.html)		

Table S2. Quality assessment of the included cohort studies

Study, year	Selection	Comparability	Outcome	Overall
Cohort studies				
Rosenberg et al, 2018	4	1	3	8
Berger et al, 2019	4	2	2	8
Krijthe et al, 2014	3	1	3	7
Magnani et al, 2014	3	1	3	7
O'Neal et al, 2017	4	2	2	8
Zeller et al, 2018	4	2	3	9
Case-control studies				
Lian, 2015	2	1	3	6
Wei, 2017	2	2	3	7
Ma, 2019	2	2	3	7
Lai et al, 2009	2	1	2	5

Table S3. Sensitivity analysis on the association of atrial fibrillation risk with total testosterone levels in men and women by omitting one study in a time

Author, Year	Men			Women		
	RR	95% CI	I^2, %	RR	95% CI	I^2, %
Berger et al, 2019	0.921	0.711-1.192	78.5	1.165	0.932-1.457	64.9
Magnani et al, 2014	1.127	0.892-1.425	82.2	--	--	--
O'Neal et al, 2017	0.952	0.707-1.283	86.6	1.105	0.927-1.317	70.2
Zeller et al, 2018	1.017	0.674-1.533	86.2	0.981	0.823-1.169	0.0

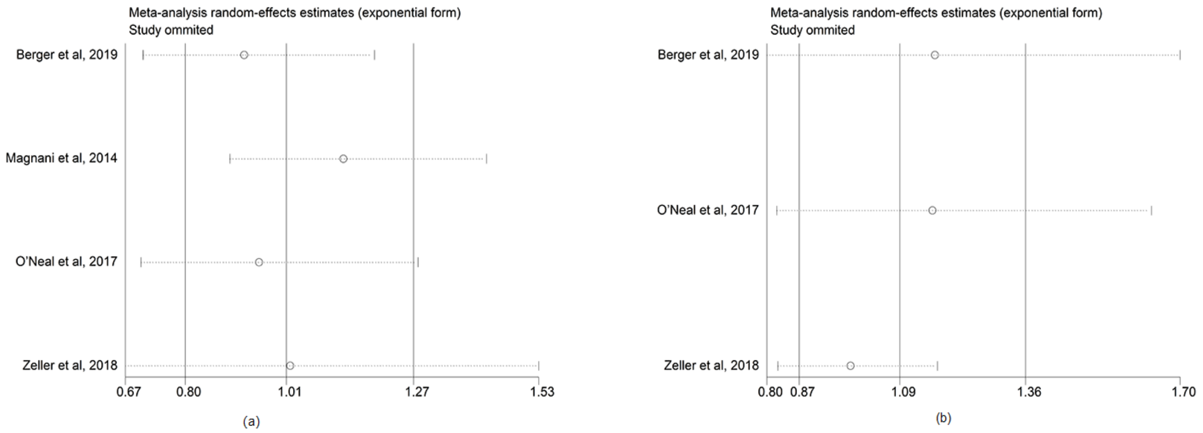

Figure S1. Sensitivity analyses on the association between levels of total testosterone and risk of atrial fibrillation (a): total testosterone level and atrial fibrillation risk in men; (b): total testosterone level and atrial fibrillation risk in post-menopausal women.
